# Circular RNAs: roles in hepatocellular carcinoma immune regulation, implications in immunotherapy, and prospects for clinical translation

**DOI:** 10.3389/fmolb.2025.1750832

**Published:** 2026-01-12

**Authors:** Yifan Chen, Jiayi Zhao, Rui Pu, Chao Fu, Zishuai Li, Jianhua Yin, Guangwen Cao

**Affiliations:** 1 Department of Epidemiology, Faculty of Navy Medicine, Second Military Medical University, Shanghai, China; 2 Key Laboratory of Biological Defense, Ministry of Education, Second Military Medical University, Shanghai, China; 3 Shanghai Key Laboratory of Medical Bioprotection, Second Military Medical University, Shanghai, China

**Keywords:** biomarker, circRNA, HCC, immune, microenviroment

## Abstract

Circular RNAs (circRNAs) are emerging as pivotal regulators within the tumor immune microenvironment (TIME) of hepatocellular carcinoma (HCC). Despite the transformative role of immunotherapy, its efficacy remains limited by primary and acquired resistance, a challenge not fully addressed by current research. This review uniquely synthesizes the latest evidence to delineate how specific circRNAs orchestrate immunosuppression in HCC through two interconnected axes: (1) by directly modulating the function and polarization of key immune cells (e.g., T cells, NK cells, macrophages), and (2) by interfering with core immune-related signaling pathways (e.g., NF-κB, MAPK, Wnt/β-catenin). We critically examine how these mechanisms collectively fuel immune evasion and confer resistance to immune checkpoint inhibitors. Moving beyond mechanism, we further explore the dual translational potential of circRNAs: as stable, minimally invasive diagnostic/prognostic biomarkers and as novel therapeutic targets via RNA interference or circRNA-based vaccine strategies. By connecting fundamental molecular insights to clinical challenges, this review provides a cohesive framework for understanding circRNA-driven immunomodulation in HCC and highlights promising avenues for overcoming immunotherapy resistance.

## 1 Introduction

Primary liver cancer (PLC) is the third leading cause of cancer-related death worldwide, imposing a substantial global health burden (Bray et al., 2024). Hepatocellular carcinoma (HCC), a malignant tumor originating from hepatocytes, accounts for 75%–85% of PLC cases globally and a higher proportion of 93% in mainland China (Lin et al., 2022; El-Serag, 2011). Clinically, HCC is asymptomatic during early disease stages, resulting in delayed diagnosis at advanced stages for most patients and a 5-year survival rate below 15% (Zeng et al., 2024; Heimbach et al., 2018). Chronic hepatitis B virus (HBV) is the predominant risk factor of HCC, contributing to 84.4% of cases in mainland China, other etiologies such as hepatitis C virus infection and non-alcoholic fatty liver disease also play important roles globally (Bray et al., 2024; Huang et al., 2021; Toh et al., 2023).

The emergence of immunotherapy has ushered in a transformative era in oncology treatment, complementing traditional methods like surgery, radiotherapy, chemotherapy, and targeted therapy, providing optimism for HCC treatment (Yau et al., 2020; Finn et al., 2025). Immunotherapeutic agents represented by immune checkpoint inhibitors (ICIs) regulate the body’s inherent immune system to break the tumor’s immune escape mechanisms, enabling the immune system to re-recognize and attack tumor cells. Numerous clinical studies have demonstrated that immunotherapy exhibits favorable efficacy in a subset of HCC patients, significantly prolonging survival and improving quality of life (Cheng et al., 2022; Finn et al., 2020). However, most patients fail to achieve sustained clinical benefit with current systemic therapies. Single ICI monoclonal antibody achieves modest response rates (approximately 15%–20%) (El-Khoueiry et al., 2017; Zhu et al., 2018). Moreover, recurrence is common even after curative-intent treatments, underscoring the limitations of current approaches and the urgent need for more effective systemic strategies (Yin et al., 2024).

Recent studies have spotlighted the pivotal role of circular RNAs (circRNAs) in orchestrating resistance to cancer immunotherapy. CircRNA, a novel class of non-coding RNA, distinguished by its unique covalently closed circular structure, exhibits greater stability compared to linear RNA and is less susceptible to degradation by exonucleases (Chen, 2020). A growing body of evidence indicates that circRNA plays a pivotal role in the initiation and progression of HCC, participating in the regulation of tumor cell proliferation, apoptosis, invasion, and metastasis through diverse and complex molecular mechanisms (Kristensen et al., 2022). Concurrently, circRNA exerts crucial functions in immunomodulation by interacting with immune cells and molecules, thereby influencing the anti-tumor immune response (Ma et al., 2024). Accumulating evidence suggests that circRNAs may mediate immunotherapy resistance by regulating the tumor immune microenvironment or immune checkpoint signaling, making them promising targets to overcome treatment failure (Zhang et al., 2024). Therefore, thorough exploration of the mechanisms underlying circRNA role in HCC immunity offers the potential to identify novel targets and strategies for HCC immunotherapy, further enhancing treatment efficacy and improving patient prognosis. In this review, we focus on how circRNAs regulate the immune response in HCC via intricate molecular mechanisms and immune-related signaling pathways. We further discuss their potential roles in driving resistance to HCC immunotherapy and, finally, outline their clinical translation prospects as biomarkers and therapeutic targets.

## 2 Biological characteristics and functions of circRNAs

CircRNAs are a special class of non-coding RNA, distinguished from linear RNAs by their unique closed-loop structure, which is not formed by chance but is produced by the back-splicing of linear precursor mRNA (Liu and Chen, 2022). During back-splicing, a downstream 5′splice donor site is reversely joined to an upstream 3′splice acceptor site, resulting in circRNAs lacking the 5′cap and 3′polyA tail typical of linear RNAs (Ashwal-Fluss et al., 2014; Zhang et al., 2020a). This unique structure confers high stability to circRNAs, making them resistant to exonuclease degradation and allowing them to persist in cells for extended periods (Chen and Yang, 2015). The functions of circRNAs are diverse and complex, with their role as miRNA sponges being of significant interest. Taking circRNA CiRS-7 as an example, it contains more than 70 binding sites for miR-7. It is highly and extensively associated with Argonaute proteins in a miR-7-dependent manner. Although circRNA is completely resistant to miRNA-mediated target destabilization, it strongly suppresses miR-7 activity, leading to increased levels of miR-7 targets (Hansen et al., 2013). Furthermore, circRNAs are involved in the regulation of gene transcription (Liu and Chen, 2022). Some circRNAs can interact with host DNA to form R-loop structures, and the formation of these structures can interfere with normal DNA replication, transcription processes, and post-damage repair mechanisms (Katrekar et al., 2022). For instance, circRHOT1 recruits the Tat-interacting protein to the NR2F6 promoter, inducing the expression of proto-oncogenes and promoting cell proliferation, migration, and invasion in HCC (Wang et al., 2019). Due to the absence of 5′cap structures and 3′poly(A) tails, circRNAs were generally regarded as non-coding RNAs with no capacity for protein coding. However, accumulating evidence from in-depth studies has revealed that a subset of circRNAs contains open reading frames and can be translated into proteins or peptides under specific conditions (Duan et al., 2022; Legnini et al., 2017; Pamudurti et al., 2017), which opening up new perspectives for our understanding of gene expression and regulatory mechanisms, and providing novel potential targets for disease diagnosis and treatment.

## 3 Microenvironment of HCC

The tumor microenvironment (TME) of HCC constitutes a complex and dynamic network orchestrated by diverse immune cells, soluble mediators, and stromal components, whose dysregulation directly dictates tumor progression and immune therapeutic responses (Xue et al., 2022; Li et al., 2021; Cadamuro et al., 2023). As the primary effector cells of adaptive immunity, CD8^+^ cytotoxic T lymphocytes are responsible for recognizing and eliminating HBV-infected hepatocytes and tumor cells by secreting perforin, granzyme, and interferon-γ (IFN-γ); however, their function is frequently impaired in HCC TME, characterized by exhaustion phenotypes (e.g., overexpression of PD-1, TIM-3, and LAG-3) induced by chronic viral antigen stimulation and immunosuppressive signals (Baudi et al., 2021; Yang et al., 2019; Arvanitakis et al., 2024). Regulatory T cells (Tregs) are abnormally enriched in HCC tissues, where they suppress CTL and natural killer (NK) cell activity through the secretion of interleukin-10 (IL-10) and transforming growth factor-β (TGF-β), while promoting immune tolerance via direct cell-cell contact mediated by CTLA-4 (Sawant et al., 2019; Yang et al., 2006). NK cells, as core components of innate immunity, exert direct anti-tumor effects without antigen priming, but their cytotoxicity is compromised by tumor-derived factors such as indoleamine 2,3-dioxygenase and prostaglandin E2 (PGE2), leading to reduced IFN-γ secretion and enhanced inhibitory receptor expression (Jing et al., 2024; Walker and Rotondo, 2004; Van Elssen et al., 2011). Tumor-associated macrophages, the most abundant immune cells in HCC TME, predominantly polarize toward the M2 phenotype under the induction of HBV-encoded proteins (e.g., HBx) and tumor-derived cytokines, thereby promoting angiogenesis, extracellular matrix remodeling, and immune escape by secreting IL-10, TGF-β, and vascular endothelial growth factor (Zhu et al., 2021). Additionally, myeloid-derived suppressor cells accumulate in HCC TME and inhibit immune cell function through multiple mechanisms, including reactive oxygen species production, arginine depletion, and nitric oxide (NO) release (Liu et al., 2025; Ohl and Tenbrock, 2018). Soluble mediators such as cytokines (IL-6, TNF-α), chemokines (CXCL12, CCL2), and immune checkpoints (PD-L1/PD-1, CTLA-4) further exacerbate the immunosuppressive state of HCC TME, forming a vicious cycle that facilitates tumor immune escape and progression (Gong et al., 2018). Collectively, the HCC TME exhibits a predominantly immunosuppressive phenotype, and targeting key components of this microenvironment has become a promising strategy for improving immunotherapeutic efficacy (Wang et al., 2021a; Qin et al., 2020).

## 4 The pivotal role of circRNAs in immune regulation of HCC

### 4.1 The impact of circRNA on immune cell function

CircRNAs have emerged as pivotal regulators in the tumor immune microenvironment of HCC. They modulate the function of various immune cells, thereby critically influencing tumor progression and the efficacy of immunotherapy. The following discussion details their roles in key immune cell populations within the HCC microenvironment.

In the immune microenvironment of HCC, T cells serve as key immune effector cells, and their functional status directly influences the immune surveillance and cytotoxic capacity of the organism against tumor cells. Studies have shown that exosomal circGSE1 derived from HCC acts as a molecular sponge for miR-324-5p, activating the TGFBR1/Smad3 axis to promote the expansion of regulatory T cells (Tregs), thereby exacerbating immunosuppression and driving tumor progression (Huang et al., 2022). Another study revealed that exosomal circCCAR1 secreted by HCC cells can be taken up by CD8^+^ T cells, where it stabilizes PD-1 protein, leading to CD8^+^ T cell exhaustion and conferring resistance to anti-PD-1 immunotherapy (Hu et al., 2023).

Macrophage polarization is regulated by multiple circRNAs. circ0003410, which is highly expressed in HCC, upregulates CCL5 by sponging miR-139-3p, thereby recruiting and polarizing macrophages toward the M2 phenotype, which accelerates HCC malignancy (Cao et al., 2022). In contrast, hsa_circ_0074854 can suppress exosome-mediated M2 polarization of macrophages by reducing the stability of human antigen R (HuR) protein, thereby inhibiting HCC migration and invasion (Wang et al., 2021c). Additionally, hsa_circ_0110,102 sponges miR-580-5p to downregulate PPARα and inhibit CCL2 secretion, subsequently suppressing the release of pro-inflammatory cytokines from macrophages via the COX-2/PGE2 pathway, exerting a tumor-suppressive effect (Wang et al., 2021b).

In the TME, NK cells are crucial for tumor immune surveillance, and NK cell dysfunction is strongly associated with the development of malignant tumors (Shimasaki et al., 2020). The regulation of NK cell function by circRNAs is a key focus in HCC immunity. Exosomal circUHRF1 derived from HCC degrades miR-449c-5p to upregulate TIM-3 expression, inhibiting the secretion of IFN-γ and TNF-α by NK cells, thereby impairing their antitumor function and contributing to anti-PD-1 therapy resistance (Zhang et al., 2020b). Another circRNA, hsa_circ_0007456, binds to miR-6852-3p to regulate intercellular adhesion molecule-1 (ICAM-1) expression, increasing NK cell-mediated cytotoxicity against HCC cells and inhibiting tumor development (Shi et al., 2021).

**FIGURE 1:**
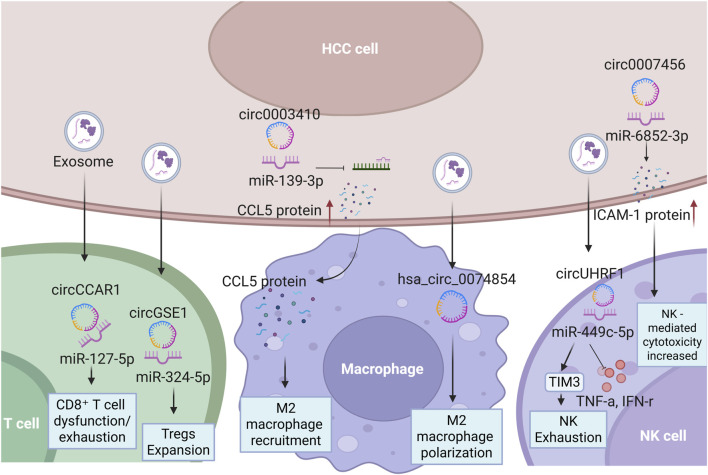
circRNA Functions in Hepatocellular Carcinoma Immune Regulation (Designed with BioRender.com) HCC, hepatocellular carcinoma; Tregs, regulatory T cells; CD8^+^ T cells, cytotoxic T lymphocytes; IFN-γ, interferon-gamma; TNF-α, tumor necrosis factor-alpha; CCL5, C-C motif chemokine ligand 5; NK Cells, natural killer cells; M2, macrophage 2.

In summary, these findings collectively underscore the critical role of circRNAs as master regulators of the immunosuppressive TME in HCC (Table 1). By exploiting exosome-mediated mechanisms, specific circRNAs orchestrate immune evasion through distinct pathways: they drive Treg expansion and impair CD8^+^ T cell function, promote the polarization of pro-tumoral M2 macrophages, and suppress the cytotoxic activity of NK cells (Figure 1). This intricate network not only facilitates HCC progression and metastasis but also contributes to resistance against current immunotherapies. Consequently, targeting these oncogenic circRNAs or their downstream pathways presents a promising strategic avenue for overcoming immunotherapy resistance and improving patient outcomes. However, there are still some limitations in existing research. Firstly, most findings are derived from cell experiments and mouse models, and their precise functions in the human tumor microenvironment require further validation through larger-scale clinical samples. Secondly, current studies often focus on the regulation of specific immune cell types by individual circRNAs, lacking a networked analysis of circRNA-mediated synergistic interactions across different immune cell subsets. For instance, it remains unknown whether the same circRNA could simultaneously regulate the functions of T cells and macrophages, potentially resulting in additive or counteractive immune effects. Additionally, discrepancies exist among different studies regarding the specific mechanisms and targets of particular circRNAs, such as hsa_circ_0074854, which may be attributed to tumor heterogeneity, variations in research models, or detection methods. Future research should employ techniques like single-cell sequencing to systematically map the circRNA-regulated immune cell interaction network in more complex *in vivo* environments and explore their potential as combinatorial therapeutic targets.

**TABLE 1:** Summary of circRNA Functions in HCC Immune Regulation.

circRNA	miRNA sponged	Immune cells affected	Impact on immunity	References
circGSE1	miR-324-5p	Tregs	Immunosuppressive: Promotes treg expansion, leading to tumor immune escape and HCC progression	PMID: 35396771
circCCAR1	miR-127-5p	CD8^+^ T cells	Immunosuppressive: Induces CD8^+^ T cell dysfunction/exhaustion and confers resistance to anti-PD1 therapy	PMID: 36932387
circ0003410	miR-139-3p	Macrophages	Immunosuppressive: CCL5 recruits and polarizes M2-type macrophages, increasing the M2/M1 ratio and promoting HCC malignancy	PMID: 34890089
hsa_circ_0074854	Not specified	Macrophages	Immunosuppressive: Induces M2 macrophage polarization, thereby promoting HCC migration and invasion	PMID: 33880025
hsa_circ_0110,102	miR-580-5p	Macrophages	Tumor-suppressive: Suppresses pro-inflammatory cytokine release from macrophages via the PPARα-CCL2 axis, inhibiting HCC development	PMID: 33891564
circUHRF1	miR-449c-5p	NK cells	Immunosuppressive: Inhibits IFN-γ and TNF-α secretion by NK cells, reduces NK cell proportion and tumor infiltration, leading to immune escape and anti-PD1 resistance	PMID: 32593303
hsa_circ_0007456	miR-6852-3p	NK cells	Tumor-suppressive: Increased the cytotoxic activity of NK cells against HCC cells, inhibiting tumor development	PMID: 33462208

circRNA, circular RNA; miRNA, microRNA; HCC, epatocellular carcinoma; Tregs, regulatory T cells; CD8^+^ T cells, cytotoxic T lymphocytes; PD1, programmed cell death protein 1; IFN-γ, interferon-gamma; TNF-α, tumor necrosis factor-alpha; CCL5, C-C motif chemokine ligand 5; PPARα, peroxisome proliferator-activated receptor alpha; NK, Cells, natural killer cells; M2, macrophage 2; M1, macrophage 1.

### 4.2 The impact of circRNA on classical immune-related signaling pathways

Beyond their roles in modulating immune cell function, circRNAs also exert profound influence on the progression and therapeutic response of HCC by directly targeting classical immune-related signaling pathways. Key pathways such as NF-κB, MAPK, and Wnt/β-catenin are frequently dysregulated in HCC and have been identified as critical downstream mechanisms through which circRNAs promote or suppress tumorigenesis.

NF-κB is a family of transcription factors that transactivate genes involved in various biological processes, including immune response, inflammation, cell growth, and survival (Mao et al., 2025). In HCC, low expression of circPTPN12 is associated with poor prognosis and promotes tumor progression by activating the NF-κB pathway, which in turn enhances the secretion of immunosuppressive cytokines such as IL-6 and TNF-α, fostering an immune-resistant microenvironment (Ji et al., 2024).

The MAPK signaling pathway is another crucial immune-related pathway, playing a key role in cell proliferation, differentiation, apoptosis, and immune responses. circDHPR acts as a ceRNA for miR-3194-5p, leading to upregulation of RASGEF1B and subsequent inhibition of the Ras/MAPK cascade, thereby suppressing tumor growth (Guo et al., 2023). In contrast, circASAP1 promotes metastasis by sponging miR-326 and miR-532-5p, which relieves their repression on MAPK1 and enhances MAPK signaling (Hu et al., 2020). Furthermore, circCCNY can bind to HSP60 and promote its ubiquitin-mediated degradation, resulting in inhibition of the MAPK pathway and restoration of lenvatinib sensitivity (Yang et al., 2025).

Dysregulation of the Wnt/β-Catenin pathway is an early event in hepatocarcinogenesis and is associated with an aggressive HCC phenotype due to its roles in cell survival, proliferation, migration, and immune evasion (Pez et al., 2013; Schwertheim et al., 2020; Yao et al., 2018). circβ-catenin encodes a novel β-catenin isoform that stabilizes full-length β-catenin and potently activates Wnt signaling, driving immune evasion and tumor progression (Liang et al., 2019). Similarly, circFADS1 facilitates GSK3β ubiquitination and degradation, leading to Wnt/β-catenin activation (Tang et al., 2025). On the other hand, circPIK3C3 functions as a tumor suppressor by sponging miR-452-5p to upregulate SOX15, which inhibits Wnt/β-catenin signaling and reverses lenvatinib resistance (Yuan et al., 2025).

**FIGURE 2:**
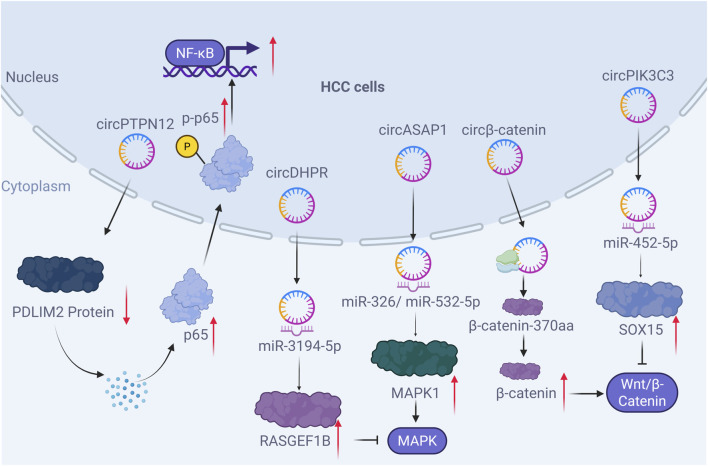
circRNAs Regulating Key Signaling Pathways in Hepatocellular Carcinoma (Designed with BioRender.com). HCC, hepatocellular carcinoma; MAPK, mitogen-activated protein kinase; NF-κB, nuclear factor kappa-B.

In conclusion, it is evident that circRNAs function as pivotal upstream regulators of several cornerstone immune signaling pathways in HCC, including NF-κB, MAPK, Wnt/β-catenin, and others (Table 2). They employ diverse mechanisms, such as acting as ceRNAs, encoding novel peptides, or facilitating protein ubiquitination, to finely tune the activity of these pathways. This intricate regulation directly impacts tumor cell proliferation, metastasis, and drug resistance (Figure 2). The accumulating evidence highlights the potential of targeting specific oncogenic circRNAs or leveraging tumor-suppressive circRNAs as a promising strategy for disrupting these vital signaling networks in HCC treatment. While existing research has successfully established a theoretical framework in which circRNAs influence the progression and therapeutic response of hepatocellular carcinoma (HCC) by regulating core signaling pathways such as NF-κB, MAPK, and Wnt/β-catenin—and has revealed diverse mechanisms including functioning as ceRNAs, encoding peptides, or modulating protein stability—the field still faces several profound challenges and limitations. A substantial body of evidence remains reliant on cell lines and mouse models, which inadequately reflect the highly heterogeneous tumor microenvironment in humans. Many mechanistic studies lack rigor, such as insufficient validation of the competitive binding conditions required for ceRNA functionality. Moreover, contradictions frequently arise across different studies regarding the function or even the directional role of the same circRNA, highlighting the context-dependent nature of its actions and the complexity introduced by tumor heterogeneity. More critically, current research predominantly focuses on linear regulatory models of individual molecules, lacking a systemic understanding of how circRNAs may collaboratively form regulatory networks to integratively shape the tumor signaling landscape and immune microenvironment. Future studies urgently need to employ single-cell multi-omics, spatial transcriptomics, and more physiologically relevant models to validate circRNA functional networks within authentic biological contexts. Furthermore, the clinical translation pathway—encompassing specific delivery, off-target effects, and feasibility of combination with existing therapies—must be rigorously evaluated for their potential as biomarkers or therapeutic targets. Only through such efforts can this promising research direction be translated into tangible clinical benefits.

**TABLE 2:** Summary of circRNAs Regulating Key Signaling Pathways in HCC.

circRNA	Signaling pathway	Effect on pathway	Mechanism of action	References
circPTPN12	NF-κB	Inhibitory	Low expression is associated with poor prognosis. RNA sequencing identified the NF-κB pathway as a major target, though the precise molecular mechanism remains unelucidated	PMID: 38992675
circDHPR	MAPK	Inhibitory	Acts as a ceRNA for miR-3194-5p, alleviating its suppression of the target gene upregulated RASGEF1B serves as a negative regulator of the Ras/MAPK pathway, thereby inhibiting its activity	PMID: 37099250
circASAP1	MAPK	Activating	Functions as a ceRNA for both miR-326 and miR-532-5p, sequestering them and preventing their repression of the common downstream target MAPK1, leading to pathway activation	PMID: 31838741
circCCNY	MAPK	Inhibitory	Binds to the HSP60 protein and recruits the E3 ubiquitin ligase SMURF1, promoting HSP60 ubiquitination and degradation. This degradation releases Raf kinase inhibitory protein, which subsequently suppresses the MAPK pathway	PMID: 39826668
circβ-catenin	Wnt/β-Catenin	Activating	Encodes a novel 370-amino acid β-catenin isoform that stabilizes full-length β-catenin by antagonizing GSK3β-induced β-catenin phosphorylation and degradation, thereby activating the Wnt pathway	PMID: 31027518
circFADS1	Wnt/β-Catenin	Activating	Induced by EIF4A3, it enhances the interaction between RNF114 and GSK3β, leading to GSK3β ubiquitination and degradation. This results in the subsequent activation of the Wnt/β-catenin pathway	PMID: 39965082
circPIK3C3	Wnt/β-Catenin	Inhibitory	Acts as a ceRNA for miR-452-5p, upregulating the expression of SOX15. SOX15 then inhibits the Wnt/β-catenin signaling pathway	PMID: 39863187

circRNA, circular RNA; MAPK, mitogen-activated protein kinase; NF-κB, nuclear factor kappa-B; ceRNA, competing endogenouse RNA.

## 5 The potential of circRNA as biomarker and therapeutic target for HCC

### 5.1 Research progress of circRNA as a diagnostic and prognostic biomarker

Due to the absence of distinct early symptoms and reliable, effective biomarkers, HCC patients are frequently diagnosed at an advanced stage, leading to a suboptimal overall survival rate (El-Serag and Rudolph, 2007). Currently employed diagnostic biomarkers, such as alpha-fetoprotein (AFP), AFP-L3, and DCP, possess limited diagnostic sensitivity and specificity (Pan et al., 2020; Yasuda et al., 2019). circRNAs, defined by their unique closed circular structure, demonstrate high stability and detectability in biological specimens including tissues, peripheral blood, and urine. They are also closely implicated in multiple biological processes of HCC, rendering them potential novel biomarkers for the early diagnosis and prognostic assessment of HCC. By virtue of their pivotal roles in the initiation and progression of HCC as well as their inherent stability, circRNAs exhibit substantial potential as biomarkers for the early diagnosis of HCC. Continuous advancements in detection technologies will empower circRNAs to exert a more prominent role in the early diagnosis of HCC, thereby enhancing the therapeutic outcomes and prognosis of patients.

circ-CDYL is specifically upregulated in both early-stage (BCLC 0-A) HCC tissues and corresponding sera. A three-factor logistic model integrating circ-CDYL and its target genes HDGF and HIF1AN discriminates early HCC from adjacent non-tumor tissue with an AUC of 0.73 (95% CI 0.65–0.80), a sensitivity of 75.4% and a specificity of 66.7%, significantly outperforming the conventional biomarker AFP (AUC 0.59). Expression levels of the three analytes are independently associated with tumor size, vascular invasion and other clinicopathological features, indicating their potential as non-invasive biomarkers for early detection of HCC (Wei et al., 2020). Zhang et al. demonstrated that circRNA_104,075 is highly expressed in both HCC tissues and serum samples. Additionally, it exhibits superior predictive performance for HCC compared to AFP, which suggests that circRNA_104,075 may serve as a more effective serum and tissue biomarker for HCC diagnosis than AFP (Zhang et al., 2018). circSMEK1 is significantly downregulated in metabolic dysfunction-associated steatohepatitis (MASH) and HCC, and its serum level can serve as a non-invasive diagnostic biomarker (AUC = 0.79). Its expression is negatively regulated by the splicing factor SF3B4. Restoring circSMEK1 expression or combining it with an IGF2 inhibitor shows promising therapeutic potential (Guo et al., 2025). In another study, through circRNA microarray screening and qRT-PCR validation, three circRNAs (hsa_circ_0000976, hsa_circ_0007750, and hsa_circ_0139,897) significantly upregulated in the plasma of HCC patients were identified, and a diagnostic model named CircPanel was constructed. This model demonstrated excellent diagnostic performance in both the training set and two independent validation sets. Its accuracy in distinguishing HCC patients from healthy controls, chronic hepatitis B patients, and cirrhosis patients (AUC values of 0.863, 0.843, and 0.864, respectively) was significantly higher than that of the traditional tumor marker AFP. Furthermore, CircPanel also exhibited remarkable diagnostic ability for small HCC (diameter ≤3 cm), AFP-negative HCC, and AFP-negative small HCC. These circRNAs showed good stability in plasma, and their levels decreased significantly after surgery, suggesting their origin from tumor tissue. Therefore, CircPanel is expected to become a novel, non-invasive, and highly accurate diagnostic tool for HCC, particularly suitable for early detection and AFP-negative patient populations (Yu et al., 2020). Similarly, another study, using microarray screening and multiple validation steps, identified three circRNAs (circ_0009582, circ_0037120, and circ_0140,117) that were significantly elevated in the plasma of patients with HBV-HCC. The constructed circRNA panel demonstrated excellent diagnostic performance in both training and validation sets, with AUC values of 0.800 and 0.857 for distinguishing HCC from chronic hepatitis or healthy controls, respectively. These values further improved to 0.988 and 0.955 when combined with AFP. Additionally, the levels of these three circRNAs decreased in postoperative plasma, and they were highly expressed in tumor tissues and positively correlated with plasma levels, indicating their tumor origin. The study suggests that this circRNA panel could serve as a potential predictive biomarker for HCC development in HBV-infected individuals, especially for early diagnosis and AFP-negative patients (Wu et al., 2020).

circRNAs are increasingly recognized as potential prognostic biomarkers. For instance, CircSETD3 (has_circRNA_0000567/hsa_circRNA_101,436) is significantly downregulated in HCC tissues and cell lines. Low expression of circSETD3 in HCC tissue significantly predicts poor prognosis and is associated with larger tumor size and poor differentiation in patients (Xu et al., 2019). In contrast, another study found that the level of circNFATC3 was positively correlated with NFATC3 mRNA, and high NFATC3 expression predicted a better prognosis; thus, circNFATC3 may be a biomarker for favorable prognosis in HCC patients (Jia et al., 2021). Furthermore, our team discovered that circKCNN2 is transcriptionally repressed by NFYA and inhibits HCC recurrence via the miR-520c-3p/MBD2 axis. The intrinsic level of circKCNN2 in HCC cells confers anti-tumor effects to lenvatinib. circKCNN2 may be a promising predictive biomarker for HCC recurrence and a potential therapeutic agent (Liu et al., 2022).

As summarized in Table 3, multiple circRNAs and circRNA panels have shown promising diagnostic and prognostic performance for HCC. Although these circRNA panels demonstrate potential that surpasses the traditional marker AFP, their clinical translation still faces significant challenges. Existing studies generally have limited sample sizes and are mostly single-center, lacking validation through prospective, multi-center, large-scale cohorts, which affects the reproducibility and general applicability of the results. The diagnostic circRNA profiles reported across different studies show low overlap, likely due to heterogeneity in patient populations (e.g., differing etiologies and stages), variations in sample processing protocols, and the lack of standardized detection technologies for circRNAs (such as primer design and normalization methods). Furthermore, most studies have not adequately assessed the specificity of these circRNA markers in distinguishing HCC from other benign liver diseases, such as hemangiomas or focal nodular hyperplasia. Moving forward, establishing standardized protocols for circRNA detection and data analysis, as well as validating them in prospective cohorts that include diverse liver disease controls, are essential steps toward advancing their clinical application.

**TABLE 3:** Summary of circular RNAs as diagnostic and prognostic biomarkers for hepatocellular carcinoma.

circRNA	Type	Group	AUC	Sensitivity	Specificity	References
Circ-CDYL	Diagnostic	Early-stage HCC vs. non-tumor tissue	0.730, superior to AFP (AUC 0.59)	75.36%	66.67%	PMID: 31148183
circRNA_104,075	Diagnostic	HCC vs. healthy	0.973, superior to AFP (AUC 0.750)	96%	98.3%	PMID: 30361504
circSMEK1	Diagnostic	HCC vs. healthy	Serum: 0.790	75%	90%	PMID: 41103217
			tissue:0.742	71%	80%	
circRNA panel (hsa_circ_0000976, hsa_circ_0007750, hsa_circ_0139,897)	Diagnostic	HCC vs. healthy	0.861, superior to AFP (AUC 0. 842)	81.60%	68.40%	PMID: 31456215
		HCC vs. CHB	0.870, superior to AFP (AUC 0. 765)			
		HCC vs. cirrhosis	0.858, superior to AFP (AUC 0. 765)			
circRNA panel (circ_0009582, circ_0037120, circ_0140,117)	Diagnostic	HCC vs. healthy	0.857, superior to AFP (AUC 0. 740)	PPV: 95%, NPV: 95%		PMID: 32692470
		HCC vs. CH	0.800	PPV: 90%, NPV: 95%		
circSETD3	Prognostic	—	—	Low circSETD3 predicts poor prognosis		PMID: 30795787
circNFATC3	Prognostic	—	—	Low circNFATC3 predicts poor prognosis		PMID: 32667692
circKCNN2	Prognostic	—	—	Low circKCNN2 predicts poor prognosis		PMID: 35051313

AUC, area under the curve; HCC, hepatocellular carcinoma; AFP, Alpha-Fetoprotein; CHB, Chronic Hepatitis B; CH, chronic hepatitis; PPV, positive predictive value; NPV, negative predictive value.

### 5.2 Exploration of circRNA-Based therapeutic strategies

Therapeutic strategies targeting circRNA are a research hotspot in the field of HCC treatment, currently focusing on RNA interference and circRNA vaccines, among others. RNA interference (RNAi) technology is an effective method for gene silencing, involving three RNA molecules (siRNA, shRNA, and antisense oligonucleotides) (Løvendorf et al., 2023; Liu et al., 2024), which can be used to target and silence circRNAs associated with the development and progression of HCC. By developing a novel siRNA delivery system based on superparamagnetic iron oxide nanoparticles, targeted silencing of circ_0058051 was effectively achieved. Experimental results demonstrated that this magnetic nanoplatform significantly inhibited HCC cell proliferation and tumor growth both *in vitro* and *in vivo*, with no apparent toxicity, exhibiting excellent targeting ability and safety. This provides a new strategy and promising clinical application prospect for gene therapy of HCC (You et al., 2023). However, RNAi molecules have limitations such as instability, low targeting efficiency, difficulties in transfection, and immunogenicity.

Due to their excellent stability and protein-coding capacity, circRNAs are considered an emerging immunotherapy strategy and show great promise in immunotherapy. circRNA vaccines, characterized by their low immunogenicity/cytotoxicity, can enhance the immune response to tumor antigens in terms of strength, breadth, quality, and duration (Niu et al., 2023; Bowen et al., 2018). Compared to traditional linear mRNA vaccines, circRNA vaccines exhibit higher stability, can persist in the body for extended periods, and continuously express antigens, thereby inducing a more durable and potent immune response. Currently, although research on circRNA vaccines in HCC is still in its early stages, some encouraging results have been achieved. Wang et al. designed and developed a circRNA vaccine platform capable of encoding the Ptpn2_I383T peptide as an HCC-specific neoantigen (Wang et al., 2024). This platform significantly activated dendritic cell maturation *in vitro* and induced T-cell activation. It demonstrated notable efficacy in both therapeutic and preventive settings in HCC mouse models. Another advantage of circRNAs in the vaccine field is their ability to activate various immune cells and influence different immune pathways. As a result, circRNAs can function as self-adjuvants for autonomous immune regulation, enhancing anti-tumor immunity, modulating the TME, improving vaccination efficiency, and reducing adverse effects associated with other immune adjuvants (Li et al., 2023). Another research developed a novel HCC neoantigen vaccine based on circRNA. Using the PIE strategy, they synthesized *in vitro* circRNA encoding tumor-specific neoantigens (e.g., the PTPN2 mutant peptide) and delivered it efficiently via lipid nanoparticles. In subcutaneous, orthotopic, and prophylactic mouse HCC models, the vaccine elicited robust tumor suppression, achieving complete regression in 60%–80% of tumors, increased infiltration of tumor-specific T cells and central memory T cells, prolonged survival, and showed no apparent toxicity. This work provides the first proof-of-concept that a circRNA neoantigen vaccine expressing endogenous tumor mutations is feasible for cancer therapy (Wang et al., 2024). Several research teams are exploring the combination of circRNA vaccines with immune checkpoint inhibitors to enhance the efficacy of immunotherapy. This combination strategy holds promise for disrupting the immune evasion mechanisms of tumors, improving the survival rate and quality of life for HCC patients, and bringing new breakthroughs in the treatment of HCC.

Although circRNA vaccines and RNAi therapies hold great promise, both are currently at the preclinical research stage and remain distant from clinical application. Key challenges lie in their *in vivo* delivery efficiency, targeting specificity, and long-term safety. For instance, achieving specific liver-targeted delivery of circRNA drugs or vaccines while avoiding off-target effects and potential immunogenicity represents a pressing technical bottleneck. Existing studies on circRNA vaccines are largely based on individual neoantigen models, and whether their efficacy applies to highly heterogeneous HCC populations still requires validation. Furthermore, combining circRNA-targeted therapies—such as silencing oncogenic circRNAs—with immunostimulatory strategies like circRNA vaccines, or integrating them with existing immune checkpoint inhibitors, may yield synergistic effects. However, the related combination strategies and their optimal sequencing regimens remain to be systematically explored. Future research should focus on developing efficient and safe delivery systems and evaluating the potential and mechanisms of combination therapies in models that more closely mimic human disease, such as humanized mouse models.

## 6 Challenges and prospects

Although circRNA research has provided new insights into understanding immune regulation in HCC and demonstrates potential diagnostic and therapeutic value, its translation from mechanistic exploration to clinical application faces multiple challenges. Current studies predominantly focus on descriptive phenotyping and preliminary mechanism validation, leaving significant limitations in methodological reliability, mechanistic depth, clinical applicability, and safety.

### 6.1 Technical challenges in detection and quantification

Current circRNA detection primarily relies on techniques such as qRT-PCR, RNA sequencing, and microarrays. However, these methods still face shortcomings in sensitivity, specificity, and quantitative accuracy. Detecting low-abundance circRNAs remains a challenge, and conventional methods struggle to accurately distinguish between highly homologous circRNA isoforms due to their overlapping sequences. Furthermore, the lack of standardized protocols for circRNA enrichment and library construction leads to poor comparability of data across different laboratories. This partially explains the low overlap observed in circRNA marker profiles from various studies. Future efforts necessitate the development of next-generation technologies, including long-read sequencing, digital detection, and single-molecule imaging, combined with optimized bioinformatics pipelines for circRNA annotation and quantification to enhance data reliability and reproducibility (Mi et al., 2022; Vromman et al., 2023).

### 6.2 Heterogeneity of HCC driven by different etiologies

HCC pathogenesis is highly heterogeneous, with major etiologies including hepatitis B virus (HBV), hepatitis C virus (HCV), and non-alcoholic steatohepatitis (NASH). The immune microenvironment, gene expression profiles, and clinical progression of HCC differ significantly across these etiologic backgrounds. Yet, most current circRNA studies do not systematically stratify patients by etiology, meaning their conclusions may only apply to specific subpopulations. Future research needs to be conducted in cohorts defined by different etiologic drivers to uncover etiology-specific patterns of circRNA expression and function, thereby promoting the development of personalized diagnosis and treatment strategies.

### 6.3 Delivery hurdles and safety concerns for circRNA-Based therapeutic strategies

circRNA-based intervention strategies (e.g., RNA interference, circRNA vaccines) have shown promise in preclinical research, but their *in vivo* delivery efficiency, targeting specificity, and stability remain major bottlenecks. circRNA molecules are relatively large, negatively charged, difficult to cross cell membranes, and susceptible to degradation by serum nucleases. Existing delivery systems (e.g., lipid nanoparticles, exosomes) require further optimization regarding liver targeting, loading efficiency, and *in vivo* distribution. Additionally, while circRNA vaccines offer advantages like high stability and prolonged expression, their potential immunogenicity cannot be ignored. Exogenous circRNA might be recognized by intracellular pattern recognition receptors, triggering non-specific innate immune responses that could compromise vaccine safety and efficacy. Future work must focus on optimizing the chemical modifications and delivery vehicles for circRNAs, alongside systematically evaluating their immune-activating properties and long-term toxicity in preclinical models.

### 6.4 Lack of network-based and dynamic perspectives in mechanistic studies

Existing research predominantly focuses on linear pathways where a single circRNA regulates downstream target genes via ceRNA mechanisms, lacking an integrated analysis of circRNA’s dynamic roles within multicellular, multi-pathway interaction networks. A single circRNA may simultaneously influence tumor cells, immune cells, and stromal cells within the same microenvironment, with its function being spatiotemporally specific and context-dependent. Moreover, research on the immunomodulatory functions of circRNA-encoded peptides is still in its infancy, and the synergistic or antagonistic relationships between this coding function and the classical non-coding roles urgently need clarification. Integrating single-cell multi-omics, spatial transcriptomics, and live-cell imaging technologies to dynamically capture circRNA functional networks in *in vivo* models will be a key focus for future mechanistic investigations.

### 6.5 Insufficient standardization and evidence strength for clinical translation

The clinical translation of circRNAs as biomarkers or therapeutic targets urgently requires standardized operational procedures and validation in large-scale, prospective cohorts. Most current diagnostic studies suffer from limited sample sizes, retrospective designs, and a lack of independent validation cohorts, undermining the credibility for clinical adoption. On the therapeutic front, circRNA-based interventions remain in the preclinical stage, with their pharmacokinetics, optimal dosing regimens, and long-term safety assessment frameworks yet to be established. Promoting multicenter collaborations, establishing standardized circRNA detection and reporting guidelines, and initiating prospective clinical studies are essential steps to bridge the gap between basic research and clinical application.

### 6.6 Future perspectives

Future circRNA research should be dedicated to constructing a translational pathway integrating “mechanism, technology, and clinic” into a cohesive framework. Mechanistically, it should leverage multi-omics and artificial intelligence approaches to unveil the dynamic regulatory networks of circRNAs within the HCC immune microenvironment. Technologically, it necessitates innovation in detection and delivery tools to enhance research precision and intervention specificity. Clinically, it should promote individualized studies guided by etiology and prognosis stratification, driving circRNA biomarkers and therapies into clinical trials. Ultimately, through multidisciplinary collaboration and industry-academia integration, circRNAs hold promise to become the next breakthrough frontier in HCC immunotherapy.
